# A Whole Genome Screen for Minisatellite Stability Genes in Stationary-Phase Yeast Cells

**DOI:** 10.1534/g3.112.005397

**Published:** 2013-04-01

**Authors:** Bonnie Alver, Peter A. Jauert, Laura Brosnan, Melissa O’Hehir, Benjamin VanderSluis, Chad L. Myers, David T. Kirkpatrick

**Affiliations:** * Department of Genetics, Cell Biology and Development, University of Minnesota, Minneapolis, Minnesota 55455; † Department of Computer Science and Engineering, University of Minnesota, Minneapolis, Minnesota 55455

**Keywords:** DNA stability, stationary phase, G_0_, quiescence

## Abstract

Repetitive elements comprise a significant portion of most eukaryotic genomes. Minisatellites, a type of repetitive element composed of repeat units 15−100 bp in length, are stable in actively dividing cells but change in composition during meiosis and in stationary-phase cells. Alterations within minisatellite tracts have been correlated with the onset of a variety of diseases, including diabetes mellitus, myoclonus epilepsy, and several types of cancer. However, little is known about the factors preventing minisatellite alterations. Previously, our laboratory developed a color segregation assay in which a minisatellite was inserted into the *ADE2* gene in the yeast *Saccharomyces cerevisiae* to monitor alteration events. We demonstrated that minisatellite alterations that occur in stationary-phase cells give rise to a specific colony morphology phenotype known as blebbing. Here, we performed a modified version of the synthetic genetic array analysis to screen for mutants that produce a blebbing phenotype. Screens were conducted using two distinctly different minisatellite tracts: the *ade2-min3* construct consisting of three identical 20-bp repeats, and the *ade2-h7.5* construct, consisting of seven-and-a-half 28-bp variable repeats. Mutations in 102 and 157 genes affect the stability of the *ade2-min3* and *ade2-h7.5* alleles, respectively. Only seven hits overlapped both screens, indicating that different factors regulate repeat stability depending upon minisatellite size and composition. Importantly, we demonstrate that mismatch repair influences the stability of the *ade2-h7.5* allele, indicating that this type of DNA repair stabilizes complex minisatellites in stationary phase cells. Our work provides insight into the factors regulating minisatellite stability.

Various types of repetitive noncoding DNA exist in abundance within eukaryotic genomes. Categorized by repeat unit size, repetitive elements consist of units that can range from one nucleotide to hundreds of nucleotides in length (reviewed in Debrauwere *et al.* 1997). Minisatellites (also known as variable number of tandem repeats) are classically defined as repetitive tracts of DNA consisting of repeat units that are specifically 15−100 bp in size (Vergnaud and Denoeud 2000). These repetitive elements are predominantly stable in actively dividing cells yet change in repeat length as well as in composition during meiosis (Jauert *et al.* 2002; Richard *et al.* 2008).

Minisatellites have been shown to perform several important biological functions. These functions include regulating gene transcription (Cohen *et al.* 1987; Krontiris 1995; Spandidos and Holmes 1987) interfering with gene splicing (Turri *et al.* 1995), acting as chromosomal fragile sites (Lukusa and Fryns 2008; Yu *et al.* 1997), and influencing chromosomal pairing during meiosis (Ashley 1994; Chandley 1989). A well-characterized example of minisatellite genomic function is that associated with the human *HRAS1* oncogene (Capon *et al.* 1983). This minisatellite is composed of nonidentical repeat units that are each 28 bp in length and have a high GC content (68%). Each repeat varies, with either a C or G at nucleotides +14 and +22 (numbered relative to the first nucleotide in the repeat). The *HRAS1* minisatellite is located 3′ of the *HRAS1* open reading frame (ORF) and acts as a binding site for the rel/nuclear factor-κB family of transcription factors (Trepicchio and Krontiris 1992). Altered minisatellites exhibit enhancement of *HRAS1* transcription (Spandidos and Holmes 1987; Krontiris 1995), indicating minisatellites can significantly influence the expression of nearby genes. Altered human minisatellites are associated with an increased risk of myoclonus epilepsy (Lafreniere *et al.* 1997; Virtaneva *et al.* 1997), diabetes mellitus (Kennedy *et al.* 1995), asthma (Kirkbride *et al.* 2001), attention deficit-hyperactivity disorder (Faraone *et al.* 2001; Yang *et al.* 2007), and several different types of cancer (Calvo *et al.* 1998; Krontiris 1995; Rosell *et al.* 1999; Vega *et al.* 2001; Weitzel *et al.* 2000). The presence of rare altered alleles of the *HRAS1* minisatellite correlates with tumors of the lung, bladder, ovaries, and brain and have been isolated from the primary tumors of patients with breast cancer (Devlin *et al.* 1993; Ding *et al.* 1999; Rosell *et al.* 1999; Vega *et al.* 2001; Weitzel *et al.* 2000).

The majority of human cells exist as a population of nondividing, quiescent cells that are contact and growth inhibited. Cancer formation in eukaryotic organisms requires loss of these inhibitory mechanisms. An initial oncogenic event can result in genomic instability within a quiescent cell, promoting uncontrolled re-entry into the cell cycle, leading to tumorigenesis (Jin *et al.* 2009; Kim *et al.* 2005; Suda *et al.* 2005). At present, little is known about genomic instability events in nondividing cells. The yeast *Saccharomyces cerevisiae* can serve as a model organism for the study of genomic instability in the context of a quiescent cell population, as yeast can enter a nonmitotic state known as stationary phase (Gray *et al.* 2004; Werner-Washburne *et al.* 1993) that mimics several key characteristics associated with mammalian quiescent G_0_ cells, including a reduced level of gene expression and condensed unreplicated chromosomes.

We previously developed a colony color segregation assay to monitor minisatellite repeat alterations in yeast (Kelly *et al.* 2007, 2011, 2012). We inserted either a minisatellite consisting of three 20-bp repeat units and a 5-bp linker (the *ade2-min3* allele) or a minisatellite consisting of seven-and-a-half variable repeats of the *HRAS1*-associated minisatellite (the *ade2-h7.5* allele) into the ORF of *ADE2* (Figure 1, A and B). These insertions create frameshifts, resulting in Ade^−^ red colonies that require supplemental adenine for growth. The red pigment is produced as a byproduct of the disruption of the adenine biosynthetic pathway (Smirnov *et al.* 1967). Loss of a repeat unit or gain of two repeat units within the minisatellite restores the *ADE2* reading frame, rendering cells white and Ade^+^. Minisatellite alterations that occur in stationary phase cells after colony formation is complete lead to the formation of white microcolonies (“blebs”) that arise on the surface of the red colony. This phenotype allows us to easily detect minisatellite alterations that occur in cells within the postmitotic cellular population.

**Figure 1:**
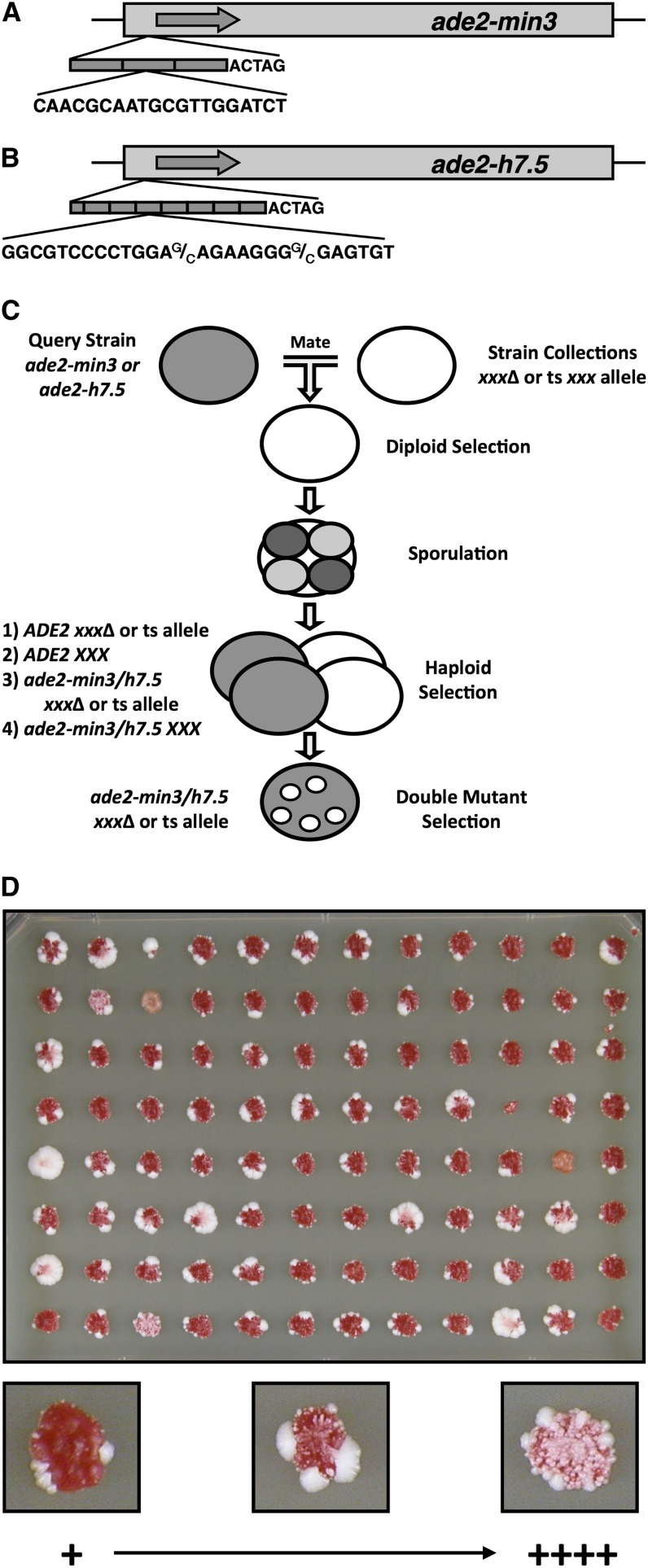
SGA analysis to screen for minisatellite instability. (A) The *ade2-min3* allele consists of three identical 20-bp repeats plus one additional base pair inserted into the *Xba*I site of *ADE2* resulting in a 5-bp overhang. Insertion of the minisatellite disrupts the *ADE2* ORF. A gain of two repeats or loss of one repeat restores the *ADE2* reading frame. (B) The *ade2-h7.5* allele contains seven-and-a-half 28-bp repeats that differ at positions +14nt and + 22nt with a C or a G as well as flanking sequence unique to the *HRAS1* locus. The minisatellite is inserted at the *Xba*I site of *ADE2* and throws the gene out of frame. Loss of a repeat restores the open reading frame. (C) Summary of the modified SGA screen. A query strain bearing the *ade2-min3* or *ade2-h7.5* allele was mated to the entire *S. cerevisiae* nonessential deletion collection or an essential strain collection containing ts mutant alleles. At different steps, strains were pinned to various selective media to isolate the desired mutant strains. Final haploid strains bearing the minisatellite allele plus a deletion or ts allele were assessed for a blebbing phenotype. (D) Each screen was performed using a 96-well format. Strains on plates from the final double mutant selection step were scored for a blebbing phenotype on a qualitative scale of + to ++++ (++++ = level of blebbing produced by the *zrt1*Δ positive control).

We previously used the *ade2-min3* reporter construct to identify mutants that increase stationary-phase minisatellite instability. An ultraviolet (UV) mutagenesis screen identified several mutations in *END3* (Kelly *et al.* 2012) and in the zinc homeostasis genes *ZRT1* and *ZAP1* (Kelly *et al.* 2007). Minisatellite alterations were specific to the quiescent population of stationary-phase cells and were independent of adenine auxotrophy or chromosomal context (Kelly *et al.* 2011). Thus, zinc homeostasis is essential for maintenance of minisatellite stability during the stationary phase possibly due to disruption of zinc-dependent DNA-binding proteins involved in DNA stability, specifically in stationary-phase cells. Minisatellite tract alterations in these mutants require homologous recombination, and multiple pathways act to maintain minisatellite stability in stationary phase cells (Kelly *et al.* 2011, 2012). Genes involved in these pathways include *END3*, *RAD27*, *PKC1*, *ZRT1*, and *ZAP1*. More recently, we used a modified version of the synthetic genetic array (SGA) protocol (Li *et al.* 2011; Tong and Boone 2006; Tong *et al.* 2001) to screen all of the genes annotated for checkpoint function and found that a subset of those genes also influence stationary phase minisatellite stability (Alver *et al.* 2013). Here, we used our modified SGA protocol to screen the entire yeast genome in an effort to: (1) determine what factors are involved in regulating minisatellite stability in stationary phase cells and (2) determine whether minisatellites varying in repeat composition and size are regulated by different factors in stationary phase cells.

## Materials and Methods

### Media and yeast strains

The solid and liquid media used in this study were prepared as stated in (Guthrie and Fink 1991). All media that was used in the SGA analysis was prepared as described in (Tong and Boone 2006; Tong *et al.* 2001) with the exception of presporulation media that contains 2% agar, 5% dextrose, 1% Difco yeast extract, and 3% Difco nutrient broth. Media consisting of Geneticin (G418) was prepared by adding 200 mg/L G418 sulfate (Cellgro).

The plasmids and *S. cerevisiae* strains used for this study are listed in Table 1. All strains, except those used to perform the SGA analyses, were derived from DTK271 (*MAT*α *his7-2 leu2*::*HisG trp1-289 ura3-52 ade2-min3*) (Kelly *et al.* 2007), in the AMY125 background. Yeast strains bearing deletions of nonessential genes were constructed by isolating genomic DNA from the G418-resistant nonessential Yeast Deletion Strain Haploid Set. Polymerase chain reaction (PCR) products containing the *KANMX4* gene (G418 resistance) and 5′ and 3′ regions of genomic homology were constructed using the primers listed in Table 2. The parental strains were transformed with the PCR product, and the transformants were selected on yeast extract-peptone-dextrose (YPD) + G418 solid media and verified by PCR.

**Table 1:** Plasmids and yeast strains used in this study

Strain	Relevant genotype	Construction details
pDC369	*URA3MX*	Gift from D. Clarke; originally from M. Tyers (p*URAMX*)
DCY2556	*his3-1 ura3-0 can1*::*MFA1pr-spHIS5*, *MAT*a	gift from D. Clarke; originally from M. Tyers (2446-14-2)
DCY2557	*his3-1 ura3-0 can1*:: *MFA1pr-spHIS5*, *MAT*α	gift from D. Clarke; originally from M. Tyers (3172-50-4)
DTK264	*ade2-min3*, *MAT*a	Kelly *et al.* 2007
DTK271	*ade2-min3*, *MAT*α	Kelly *et al.* 2007
DTK878	*ade2-min3*, *zrt1*Δ::*KANMX*	Kelly *et al.* 2007
DTK893	*ade2-min3 - URA3MX his3-1 ura3-0 can1*::*MFA1pr-spHIS5*, *MAT*a	Alver *et al.* 2013
DTK902	*ade2-min3 zap1*Δ	Kelly *et al.* 2007
DTK1082	*ade2-min3*, *cot1*Δ::*KANMX*	Kelly *et al.* 2011
DTK1175	*ade2-min3 - URA3MX his3-1 ura3-0 can1*:: *MFA1pr-spHIS5*	Alver *et al.* 2013
DTK1188	*ade2-h7.5*	Kelly *et al.* 2011
DTK1189 2b	*ade2-min3 - URA3MX his3-1 ura3-0 can1*:: *MFA1pr-spHIS5*, *MAT*α	Alver *et al.* 2013
DTK1189 5a	*ade2-min3 - URA3MX his3-1 ura3-0 can1*:: *MFA1pr-spHIS5*, *MAT*a	Alver *et al.* 2013
DTK1200	*ade2-h7.5 zrt1*Δ	Kelly *et al.* 2011
DTK1624	*ade2-h7.5 - URA3MX his3-1 ura3-0 can1*:: *MFA1pr-spHIS5*, *MAT*a	DCY2556 with *ade2-h7.5 - URA3MX* linked cassette
DTK1699	*ade2-min3*, *mon1*Δ::*KANMX*	DTK271 with *mon1*Δ::*KANMX**^a^*
DTK1900	*ade2-h7.5*, *mlh1*Δ::*KANMX*	DTK1188 with *mlh1*Δ::*KANMX**^a^*
DTK1901	*ade2-h7.5*, *mlh3*Δ::*KANMX*	DTK1188 with *mlh3*Δ::*KANMX**^a^*
DTK1902	*ade2-h7.5*, *pms1*Δ::*KANMX*	DTK1188 with *pms1*Δ::*KANMX**^a^*
DTK1903	*ade2-h7.5*, *msh2*Δ::*KANMX*	DTK1188 with *msh2*Δ::*KANMX**^a^*
DTK1904	*ade2-h7.5*, *mlh3*Δ::*KANMX*	DTK1188 with *mlh3*Δ::*KANMX**^a^*
DTK1905	*ade2-h7.5*, *mlh2*Δ::*KANMX*	DTK1188 with *mlh2*Δ::*KANMX**^a^*
DTK1906	*ade2-h7.5*, *exo1*Δ::*KANMX*	DTK1188 with *exo1*Δ::*KANMX**^a^*
DTK1907	*ade2-h7.5*, *msh6*Δ::*KANMX*	DTK1188 with *msh6*Δ::*KANMX**^a^*
DTK1920	*ade2-min3*, *mrc1*Δ::*KANMX*	DTK1657 with *mrc1*Δ::KANMX*^a^*
DTK1973	*ade2-h7.5*, *cot1*Δ::*KANMX*	DTK1188 with *cot1*Δ::*KANMX**^a^*
DTK1975	*ade2-h7.5*, *zap1*Δ::*KANMX*	DTK1188 with *zap1*Δ::*KANMX**^a^*
DTK1990	*ade2-h7.5*, *mon1*Δ::*KANMX*	DTK1188 with *mon1*Δ::*KANMX**^a^*
DTK1991	*ade2-min3*, *rpl22a*Δ::*KANMX*	DTK271 with *rpl22a*Δ::*KANMX**^a^*
DTK1992	*ade2-h7.5*, *rpl22a*Δ::*KANMX*	DTK1188 with *rpl22a*Δ::*KANMX**^a^*
DTK1993	*ade2-min3*, YGL217CΔ::*KANMX*	DTK271 with YGL217CΔ::*KANMX**^a^*
DTK1994	*ade2-h7.5*, YGL217CΔ::*KANMX*	DTK1188 with YGL217CΔ::*KANMX**^a^*

a Indicates that the strain was made using a polymerase chain reaction−generated construct.

**Table 2:** Primers

Primer	Reference number	Sequence
ADE2 F	14193008	GGGTTAGCTATTTCGCCCAATG
ADE2 F + TAG	14193006	TCCAGTTTAAACGAGCTCGAATTCGAAGCCGAGAATTTTGTAACACC
ADE2 R	14193007	TCGCCTTAAGTTGAACGGAGTC
EXO1 F	48201622	CGTCTTTAGCAAAGGCGGGAAGTA
EXO1 R	48201623	GCATTGTTCATAGCGGGGCAAA
MLH1 F	36803545	CGGTGTTTAGTAATCGCGCTAGCA
MLH1 R	36803546	CTCGGGTCTTTGGTACCGTTGAAT
MLH2 F	49451839	GCTATATTGCCCTGGCACAATG
MLH2 R	49451840	TGCAACCTCACAGAATCAGAT
MLH3 F	49451841	GCGCAAATTCAACCCCATTGAT
MLH3 R	49451842	CGGTAATGCAACAGTGGAGCAGT
MON1 F	56866461	GGCTAGTATGCGTACCTTTATCCC
MON1 R	56866462	GTGTTTGGTTAACACCCCTTCT
MSH2 F	48201624	CGCACTCCATCAAGTGAACCTCAA
MSH2 R	48201625	CCGGAGATACTCTTTCCAGTGGT
MSH3 F	48774856	AGTGTTTCCCCGACTCACCTTT
MSH3 R	48774857	TGTACAAGGCCAAGGCATAACAGT
MSH6 F	48774858	AATAAACGCGTGAGCAGTAGCTGA
MSH6 R	48774859	CTTGCCCAAGATGCGGTAAAAGA
PMS1 F	2694460	TAGAAAGCACAGATTAATAC
PMS1 R	2694461	ACATATATCCATCAAGCATC
RPL22A F	88529802	TTTTCCTTTCCACCTCAGTGCG
RPL22A R	88529803	GGCAAAGCGTCTCATAAGCAAC
YGL217C F	88529806	TGAAGGTGTGCCACTCACAGTA
YGL217C F	88529807	TCCCTTAGCTAGCCGTGTTT
ZAP1 F	14767981	ACTTGCCGCCTACTTGGC
ZAP1 R	14767982	AATGTCCTTCCCCCCCAC

We constructed the *ade2-min3* query strain DTK893 (*MAT*a *his3-1 ura3-0 can1*::*MFA1pr-spHIS5 ade2-min3 - URA3MX*) using a two-step PCR process as previously described (Alver *et al.* 2013). To summarize, the plasmid pDC369 was used to generate a *URA3MX* PCR product with flanking sequence using primers 14193004 and 14193005. DTK271 genomic DNA was used as a PCR template to isolate the *ade2-min3* allele using primers 14193006 and 14193007. To combine the *URA3MX* and *ade2-min3* PCR products, we performed PCR by using primers 14193007 and 14193008 resulting in the *ade2-min3 URA3MX*-linked cassette that was transformed into DCY2556. Transformants were selected on solid synthetic media lacking uracil (SD-Ura), resulting in DTK893. DTK893 and DCY2557 were mated, resulting in DTK1175, which was sporulated and dissected. Mating, sporulation, and tetrad dissection were performed as described previously (Jauert *et al.* 2002). Red spores were selected on solid SD-Ura media. Query strains for the SGA analyses were isolates of DTK1175: DTK1189 5a = *MAT*a derivative for the nonessential SGA and DTK1189 2b = *MAT*α derivate for the essential SGA. pDC369, DCY2556, and DCY2557 are from Dr. Duncan Clarke, University of Minnesota.

To construct the *ade2-h7.5* query strain, DTK1624 (*MAT*a *his3-1 ura3-0 can1*::*MFA1pr-spHIS5 ade2-h7.5 - URA3MX*), we generated an *ade2-h7.5 URA3MX*-linked cassette by using a two-step PCR process. In summary, we obtained a *URA3MX* PCR product bearing a 5′ TEF promoter site and a 3′ TEF terminator site from pDC369 by using primers 14193004 and 14193005. We isolated an *ade2-h7.5* PCR product from DTK1188 genomic DNA by using primers 14193006 and 14193007. The two PCR products (*URA3MX* and *ade2-h7.5*) were combined by using primers 14193007 and 14193008. The complete *ade2-h7.5 URA3MX*-linked cassette was transformed into DCY2556. Red Ura^+^ cells were selected on SD-Ura solid synthetic media, yielding DTK1624.

### SGA analysis

#### Nonessential SGA:

For our study, we performed a modified SGA analysis as described in our previous work (Alver *et al.* 2013; Tong and Boone 2006; Tong *et al.* 2001) (Figure 1C). To summarize, we inoculated YPD liquid media with a single red colony of query strain DTK1189 5a or DTK1624. Cultures were plated onto YPD solid media after overnight incubation at 30°. The *MAT*α nonessential Yeast Deletion Strain Haploid Set (Invitrogen; from Dr. Robin Wright, University of Minnesota) was pinned onto the query strain in a 96-well format (Figure 1D). Mated strains were incubated at 30° overnight. The resulting zygotes were pinned to SD-Ura + G418 solid media and incubated at 30° overnight. Diploids were then pinned to presporulation solid media, incubated at 30° overnight, and then pinned to sporulation media and incubated at room temperature (RT) for 6 d. Haploid *MAT*a progeny were selected on synthetic media lacking histidine, arginine, and uracil (SD-His/Arg/Ura) and containing canavanine (US Biological). Strains were incubated at 30° overnight. This step was repeated. Strains were then pinned to SD-His/Arg/Ura + canavanine + G418 media, and this step was repeated. The haploids were then pinned to YPD and left at RT for 5 d. Each plate was pinned in duplicate, and the screen was repeated three independent times. A positive control consisting of a strong blebbing strain (a *MAT*α *zrt1*Δ mutant; SCD153) was included on each plate (Kelly *et al.* 2007). The blebbing produced by the resulting double mutants was rated on a scale of + to ++++ (Figure 1D). Candidate hits were characterized as strains producing a strong degree of blebbing (+++ or ++++). The blebbing phenotype for each hit was verified by restreaking the strain onto YPD and assessing the individual yeast colonies for a blebbing phenotype on a scale of + to ++++.

Scores for the blebbing phenotypic analysis of the *ade2-min3* and *ade2-h7.5* nonessential SGA independent screens are listed in Supporting Information, File S1 and File S2, respectively. Duplicate plates from each screen are indicated by an “a” or “b” for each individual screen. The scores are represented numerically instead of by “+’s.” For example, a “1” indicates that a specific ORF was assigned of score of “+” for a specific screen. A score of “0” indicates that a specific strain did not grow, and a score of “5” indicates that a strain was composed of white cells and therefore could not be assigned a rating for a blebbing phenotype. A hit was defined as a strain (corresponding to a specific ORF) that produced a level of blebbing of “3−4” (+++ to ++++) in both replicates for at least two of the three independent screens performed. A second criteria used to define a hit was a strain that was scored as a “3” or “4” (+++ or ++++) in both replicates for blebbing in combination with a score of “0” (no growth) or a “5” (white patch) for a least two of the three independent screens.

#### Essential SGA:

We followed a similar screening protocol as described previously and in our previous work (Alver *et al.* 2013). In summary, the query strain DTK1189 2b was mated to a *MAT*a essential temperature-sensitive (ts) strain set containing 455 ts genes (from Dr. Charles Boone, University of Toronto) (Li *et al.* 2011) and incubated at RT for 2 d. Zygote selection, presporulation, and sporulation were performed as described previously with the exception that strains were incubated at RT. Haploid and double mutant selection were as described previously with strain incubation being carried out at RT for 2 d. After selection, the double mutant haploids bearing the *ade2-min3* allele were pinned to five separate YPD solid media plates, and the plates were incubated for 5 d at a range of temperatures: 26°, 30°, 32°, 34°, or 37°. Blebbing was scored as described previously. Strains producing a +++ to ++++ level of blebbing were restruck onto YPD and incubated at the corresponding temperature to verify the blebbing phenotype. Restruck strains were again rated on a scale of + to ++++. As noted previoulsy, a *MAT*a *zrt1*Δ haploid mutant (SCD202) was used as a positive control (Kelly *et al.* 2007).

Phenotypic scoring for each independent screen is shown in File S3. As described previously, the scores are represented numerically rather than by “+s.” Each score represents the maximum level of blebbing assigned to a specific ORF. Specifically, all of the scores assigned to each individual ts allele of a particular ORF were compared at each individual temperature. The highest blebbing score of all of the alleles and all of the incubation temperatures was assigned to the corresponding ORF for each independent screen. As described previoulsy, a hit was defined as an ORF that produced a level of blebbing of “3−4” (+++ to ++++) for at least two of the three independent screens performed or as an ORF that produced a level of blebbing of “3” or “4” (+++ or ++++) in combination with a score of “0” (no growth) or “5” (solid white patch) for at least two out of the three independent screens.

### Gene Ontology (GO) term analysis

GO data used ontology version 1.2 with annotations downloaded on January 22, 2012. Enrichment calculations are based on a hyper-geometric overlap test and reported *P*-values have been Bonferroni-corrected for multiple hypotheses (Boyle *et al.* 2004).

### Blebbing quantification assay

Yeast strains used for the quantification assay were streaked onto solid YPD media and incubated at 30° for 2 d. A single red colony was used to inoculate 5 mL of liquid YPD media, and the cultures were incubated at 30° for 4 h. Cultures were diluted and plated onto solid YPD media. Strains were incubated at 30° for 2 d and then left at RT for 6 d to allow for bleb formation. For each strain, blebs were counted on at least 100 colonies ranging in size from 1.26 to 1.32 mm in diameter. This assay was repeated three independent times whereupon the average number of blebs per colony as well as the 95% confidence interval of the mean was calculated.

## Results

### Genome-wide identification of factors required for maintaining ade2-min3 minisatellite stability in stationary-phase cells

We previously reported a unique color segregation assay that allows us to monitor minisatellite instability occurring in *S. cerevisiae* cells (Kelly *et al.* 2007, 2011, 2012). This assay employs the *ade2-min3* allele, which consists of a short minisatellite featuring three identical 20-bp repeat units inserted into the gene *ADE2* (Figure 1A). Insertion of the minisatellite plus a 5-bp overhang disrupts the *ADE2* ORF and results in red colony color. The gain of either two repeat units or the loss of one repeat unit restores the correct reading frame and results in the formation of white cells.

Our assay allows us to distinguish between minisatellite alterations that take place during mitotic growth and those that occur during the stationary phase. Alterations occurring in actively dividing cells lead to a red/white sectoring color segregation phenotype, whereas those occurring in stationary-phase cells result in a novel phenotype known as blebbing (Kelly *et al.* 2007) in which white microcolonies form on the surface of a red colony. This phenotype allows us to directly identify factors that regulate the stability of a minisatellite in a postmitotic population of yeast cells. We recently used a modification of the SGA protocol to examine genes annotated as having a checkpoint function in yeast and determined that a subset of these proteins affect the stability of the *ade2-min3* minisatellite tract in stationary-phase cells (Alver *et al.* 2013). This pilot study showed that an automated approach was not possible, as any white Ade^+^ cells that arose during the procedure rapidly overwhelmed the red Ade^−^ cells. To compensate, we modified the SGA protocols to manually screen for mutants that produced a blebbing phenotype (Figure 1C) (Alver *et al.* 2013; Li *et al.* 2011; Tong and Boone 2006; Tong *et al.* 2001).

Using the *ade2-min3* reporter, we identified 102 candidate genes that, when mutated, resulted in a strong blebbing phenotype (Table 3). These included the zinc homeostasis genes *ZRT1* and *ZAP1* identified in our original UV mutagenesis screen (Kelly *et al.* 2007), demonstrating that our SGA analysis was working correctly. Also included were the genes *PKC1*, an essential gene that encodes for a serine/threonine kinase that regulates cell wall modifications (Levin *et al.* 1990), and *RAD27*, which encodes for a nuclease that processes Okazaki fragments (reviewed in Liu *et al.* 2004a,b). Both genes were shown by our laboratory to be important for maintaining minisatellite stability in stationary phase cells (Kelly *et al.* 2012). Finally, we previously identified checkpoint-related genes including *MRC1*, *CSM3*, and *TOF1* by SGA analysis (Alver *et al.* 2013); those genes are marked in Table 3.

**Table 3:** Summary of hits from the *ade2-min3* SGA analysis of the yeast nonessential and essential strain sets

Gene	ORF	Gene	ORF	Gene	ORF	Gene	ORF	Gene	ORF
*ABD1**^a^*	YBR236C	*ERG10**^a^*	YPL028w	*ORC2**^a^*	YBR060c	*RRS1**^a^*	YOR294w	*TOF1**^b^*	YNL273w
*ADE12*	YNL220W	*FAL1**^a^*	YDR021w	*ORC3**^a^*	YLL004w	*RSP5**^a^*	YER125w	*TSC11**^a^*	YER093c
*APC11**^a^*	YDL008W	*GAS2*	YLR343w	*PAC10*	YGR078c	***SAC1***	**YKL212w**	*UPS2*	YLR168c
*ARL1*	YBR164c	*HBT1*	YDL223c	*PDS1**^a^*	YDR113c	*SEC17**^a^*	YBL050w	*URA7*	YBL039c
*ASK1**^a^*	YKL052c	*IPL1**^a^*^,^*^b^*	YPL209c	*PEP5*	YMR231w	*SEC22**^a^*	YLR268w	*USO1**^a^*	YDL058w
*ATG3*	YNR007c	*KAP122*	YGL016w	*PKC1**^a^*	YBL105c	*SEC59**^a^*	YMR013c	*VPS41*	YDR080w
*BAP3*	YDR046c	*LCB1**^a^*	YMR296c	*POB3**^a^*	YML069w	*SLA1*	YBL007c	*YCF1*	YDR135c
*BUB3**^b^*	YOR026W	*MAK10*	YEL053c	*POL31**^a^*	YJR006w	*SLI15**^a^*^,^*^b^*	YBR156c	YCL060C	YCL060C
*BUD28*	YLR062c	*MCD1**^a^*	YDL003w	*POL32*	YJR043c	*SMC5**^a^*	YOL034w	YCL075W	YCL075w
*CBF2**^a^*	YGR140W	*MCM5**^a^*	YLR274w	*PSE1**^a^*	YMR308c	*SNU114**^a^*	YKL173w	YGL114W	YGL114w
*CCZ1*	YBR131W	*MET30**^a^*	YIL046w	*RAD27*	YKL113c	*SPC29**^a^*	YPL124w	YGL217C	YGL217c
*CEP3**^a^*^,^*^b^*	YMR168c	*MFA1*	YDR461w	*RAV1*	YJR033c	*SSA1*	YAL005c	YGR291C	YGR291c
*COF1**^a^*	YLL050c	*MGA2*	YIR033w	*RFC2**^a^*	YJR068w	*SSD1*	YDR293c	YKR035C	YKR035C
*COP1**^a^*	YDL145c	*MMR1*	YLR190w	*RFC4**^a^*^,^*^b^*	YOL094c	*STT4**^a^*	YLR305c	YLR125W	YLR125w
*COT1*	YOR316c	*MMS21**^a^*	YEL019c	*RIC1*	YLR039C	*STU1**^a^*	YBL034c	YOR008C*^a^*	YOR008c
*CSM3**^b^*	YMR048w	*MOB2**^a^*	YFL034c-b	*RMD1*	YDL001w	*SWH1*	YAR042w	*YPT31*	YER031c
*DBF2*	YGR092w	*MON1*	YGL124c	*RPB3**^a^*	YIL021w	*TAF12**^a^*	YDR145w	*ZAP1*	YJL056c
*DCG1*	YIR030c	*MRC1**^b^*	YCL061c	*RPL22A*	YLR061w	*TEC1*	YBR083w	***ZRT1***	**YGL255w**
*DPB3*	YBR278w	*MUP1*	YGR055w	*RPT4**^a^*	YOR259c	*TEM1**^a^*	YML064c		
*DPB4*	YDR121w	*NNF1**^a^*	YJR112w	*RPT6**^a^*	YGL048c	*TIM22**^a^*	YDL217c		
*ERG8**^a^*	YMR220w	***ODC1***	**YPL134c**	*RRI1*	YDL216c	*TLG2*	YOL018c		

Genes appearing in bold typeface are the strongest hits from the *ade2-min3* screens (scored as ++++ for at least two of three independent screens). ORF, open reading frame.

a Genes are hits from the ts essential allele collection.

b Genes were previously reported in Alver *et al.* 2013.

To determine whether any enriched GO terms were represented within the 102 candidate hits, we performed GO term analysis using Ontology version 1.2. We find that genes associated with GO terms representing chromosomal maintenance and DNA replication are overrepresented within our list of 102 hits (Table 4), indicating these cellular processes are likely to be important for regulating the stationary phase stability of the *ade2-min3* minisatellite.

**Table 4:** Enriched GO terms of hits from the *ade2-min3* SGA analysis of the yeast nonessential and essential strain sets

GO ID	GO term	*P* value	Genes
GO:0006261	DNA-dependent DNA replication	2.25E-08	*ORC2*, *DPB3*, *MRC1*, *DPB4*, *POL31*, *POL32*, *RFC2*, *RAD27*, *ORC3*, *MCM5*, *POB3*, *CSM3*, *TOF1*, *RFC4*
GO:0005657	Replication fork	6.86E-08	*DPB3*, *MRC1*, *DPB4*, *POL31*, *POL32*, *RFC2*, *MCM5*, *POB3*, *CSM3*, *TOF1*, *RFC4*
GO:0044427	Chromosomal part	1.26E-07	*STU1*, *ORC2*, *SLI15*, *DPB3*, *MRC1*, *MCD1*, *DPB4*, *MMS21*, *CBF2*, *POL31*, *POL32*, *RFC2*, *NNF1*, *ASK1*, *ORC3*, *MCM5*, *POB3*, *CSM3*, *CEP3*, *TOF1*, *SMC5*, *RFC4*, *BUB3*, *IPL1*
GO:0043234	Protein complex	2.15E-07	*SSA1*, *STU1*, *SEC17*, *ORC2*, *SLI15*, *ABD1*, *DPB3*, *MRC1*, *MCD1*, *APC11*, *COP1*, *RRI1*, *TIM22*, *VPS41*, *DPB4*, *TAF12*, *MMS21*, *MAK10*, *TSC11*, *RSP5*, *KAP122*, *RPT6*, *PAC10*, *CBF2*, *RPB3*, *MET30*, *POL31*, *RAV1*, *POL32*, *RFC2*, *NNF1*, *ASK1*, *SAC1*, *ORC3*, *RIC1*, *SEC22*, *MCM5*, *POB3*, *CSM3*, *CEP3*, *PEP5*, *LCB1*, *TOF1*, *TLG2*, *SMC5*, *RFC4*, *BUB3*, *RPT4*, *IPL1*
GO:0005694	Chromosome	1.20E-06	*STU1*, *ORC2*, *SLI15*, *DPB3*, *MRC1*, *MCD1*, *DPB4*, *MMS21*, *CBF2*, *POL31*, *POL32*, *RFC2*, *NNF1*, *ASK1*, *ORC3*, *MCM5*, *POB3*, *CSM3*, *CEP3*, *TOF1*, *SMC5*, *RFC4*, *BUB3*, *IPL1*
GO:0006260	DNA replication	2.60E-06	*ORC2*, *DPB3*, *MRC1*, *DPB4*, *POL31*, *POL32*, *RFC2*, *RAD27*, *ORC3*, *MCM5*, *POB3*, *CSM3*, *TOF1*, *RFC4*
GO:0043596	Nuclear replication fork	3.85E-06	*DPB3*, *MRC1*, *DPB4*, *POL31*, *POL32*, *MCM5*, *POB3*, *CSM3*, *TOF1*
GO:0006272	Leading strand elongation	1.03E-05	*DPB3*, *DPB4*, *POL31*, *POL32*, *RFC2*, *RFC4*
GO:0044422	Organelle part	3.10E-05	*SSA1*, *SWH1*, *SLA1*, *STU1*, *ORC2*, *CCZ1*, *SLI15*, *ARL1*, *ABD1*, *DPB3*, *MRC1*, *YCL075W*, *MCD1*, *APC11*, *USO1*, *COP1*, *RRI1*, *TIM22*, *FAL1*, *VPS41*, *PDS1*, *DPB4*, *YCF1*, *TAF12*, *MMS21*, *YPT31*, *TSC11*, *RSP5*, *KAP122*, *MON1*, *DBF2*, *CBF2*, *RPB3*, *MET30*, *MGA2*, *POL31*, *POL32*, *RFC2*, *NNF1*, *ASK1*, *RAD27*, *SNU114*, *SAC1*, *ORC3*, *COF1*, *RIC1*, *RPL22A*, *UPS2*, *MMR1*, *SEC22*, *MCM5*, *TEM1*, *POB3*, *SEC59*, *CSM3*, *CEP3*, *PEP5*, *LCB1*, *TOF1*, *TLG2*, *SMC5*, *RFC4*, *BUB3*, *RRS1*, *COT1*, *SPC29*, *ODC1*, *IPL1*
GO:0044446	Intracellular organelle part	3.10E-05	*SSA1*, *SWH1*, *SLA1*, *STU1*, *ORC2*, *CCZ1*, *SLI15*, *ARL1*, *ABD1*, *DPB3*, *MRC1*, *YCL075W*, *MCD1*, *APC11*, *USO1*, *COP1*, *RRI1*, *TIM22*, *FAL1*, *VPS41*, *PDS1*, *DPB4*, *YCF1*, *TAF12*, *MMS21*, *YPT31*, *TSC11*, *RSP5*, *KAP122*, *MON1*, *DBF2*, *CBF2*, *RPB3*, *MET30*, *MGA2*, *POL31*, *POL32*, *RFC2*, *NNF1*, *ASK1*, *RAD27*, *SNU114*, *SAC1*, *ORC3*, *COF1*, *RIC1*, *RPL22A*, *UPS2*, *MMR1*, *SEC22*, *MCM5*, *TEM1*, *POB3*, *SEC59*, *CSM3*, *CEP3*, *PEP5*, *LCB1*, *TOF1*, *TLG2*, *SMC5*, *RFC4*, *BUB3*, *RRS1*, *COT1*, *SPC29*, *ODC1*, *IPL1*
GO:0044454	Nuclear chromosome part	3.45E-05	*ORC2*, *DPB3*, *MRC1*, *MCD1*, *DPB4*, *CBF2*, *POL31*, *POL32*, *NNF1*, *ASK1*, *ORC3*, *MCM5*, *POB3*, *CSM3*, *CEP3*, *TOF1*, *BUB3*, *IPL1*
GO:0032991	Macromolecular complex	1.28E-04	*SSA1*, *STU1*, *SEC17*, *ORC2*, *SLI15*, *ABD1*, *DPB3*, *MRC1*, *MCD1*, *APC11*, *COP1*, *RRI1*, *TIM22*, *VPS41*, *DPB4*, *TAF12*, *SSD1*, *MMS21*, *MAK10*, *TSC11*, *RSP5*, *KAP122*, *RPT6*, *PAC10*, *CBF2*, *RPB3*, *MET30*, *POL31*, *RAV1*, *POL32*, *RFC2*, *NNF1*, *ASK1*, *SNU114*, *SAC1*, *ORC3*, *RIC1*, *RPL22A*, *SEC22*, *MCM5*, *POB3*, *CSM3*, *CEP3*, *PEP5*, *LCB1*, *TOF1*, *TLG2*, *SMC5*, *RFC4*, *BUB3*, *RPT4*, *RRS1*, *IPL1*
GO:0000228	Nuclear chromosome	1.90E-04	*ORC2*, *DPB3*, *MRC1*, *MCD1*, *DPB4*, *CBF2*, *POL31*, *POL32*, *NNF1*, *ASK1*, *ORC3*, *MCM5*, *POB3*, *CSM3*, *CEP3*, *TOF1*, *BUB3*, *IPL1*
GO:0005634	Nucleus	4.64E-04	*SSA1*, *SWH1*, *SLA1*, *STU1*, *PKC1*, *ORC2*, *TEC1*, *SLI15*, *ABD1*, *DPB3*, *MRC1*, *YCL075W*, *MCD1*, *APC11*, *RRI1*, *FAL1*, *PDS1*, *DPB4*, *TAF12*, *SSD1*, *MMS21*, *RSP5*, *MOB2*, *KAP122*, *RPT6*, *CBF2*, *RPB3*, *MET30*, *MGA2*, *ZAP1*, *POL31*, *POL32*, *RFC2*, *NNF1*, *ASK1*, *RAD27*, *SNU114*, *ORC3*, *COF1*, *RIC1*, *MCM5*, *POB3*, *CSM3*, *CEP3*, *ERG8*, *PSE1*, *TOF1*, *SMC5*, *RFC4*, *BUB3*, *RPT4*, *RRS1*, *SPC29*, *IPL1*
GO:0071842	Cellular component organization at cellular level	4.70E-04	*SSA1*, *SLA1*, *STU1*, *SEC17*, *PKC1*, *ORC2*, *CCZ1*, *MRC1*, *MCD1*, *APC11*, *USO1*, *TIM22*, *VPS41*, *PDS1*, *TAF12*, *SSD1*, *TSC11*, *RSP5*, *MOB2*, *RPT6*, *PAC10*, *DBF2*, *CBF2*, *RFC2*, *NNF1*, *ASK1*, *SNU114*, *ORC3*, *COF1*, *UPS2*, *MMR1*, *SEC22*, *MCM5*, *GAS2*, *TEM1*, *POB3*, *CSM3*, *CEP3*, *PEP5*, *TOF1*, *ATG3*, *TLG2*, *RFC4*, *RPT4*, *SPC29*, *IPL1*
GO:0006271	DNA strand elongation involved in DNA replication	4.86E-04	*DPB3*, *DPB4*, *POL31*, *POL32*, *RFC2*, *RAD27*, *RFC4*
GO:0007059	Chromosome segregation	4.98E-04	*SLI15*, *MRC1*, *MCD1*, *PDS1*, *CBF2*, *RFC2*, *NNF1*, *ASK1*, *CSM3*, *TOF1*, *SMC5*, *RFC4*, *IPL1*
GO:0000793	Condensed chromosome	5.66E-04	*STU1*, *SLI15*, *MCD1*, *MMS21*, *CBF2*, *NNF1*, *ASK1*, *CEP3*, *SMC5*, *BUB3*, *IPL1*
GO:0032993	Protein−DNA complex	6.30E-04	*ORC2*, *DPB3*, *DPB4*, *POL31*, *POL32*, *ORC3*, *MCM5*, *POB3*
GO:0009987	Cellular process	6.65E-04	*SSA1*, *SWH1*, *SLA1*, *STU1*, *URA7*, *SEC17*, *PKC1*, *ORC2*, *TEC1*, *CCZ1*, *SLI15*, *ARL1*, *ABD1*, *DPB3*, *MRC1*, *YCL075W*, *RMD1*, *MCD1*, *APC11*, *USO1*, *COP1*, *RRI1*, *TIM22*, *HBT1*, *FAL1*, *BAP3*, *VPS41*, *PDS1*, *DPB4*, *YCF1*, *TAF12*, *SSD1*, *MFA1*, *MMS21*, *MAK10*, *YPT31*, *TSC11*, *RSP5*, *MOB2*, *KAP122*, *RPT6*, *YGL114W*, *MON1*, *ZRT1*, *MUP1*, *PAC10*, *DBF2*, *CBF2*, *RPB3*, *MET30*, *MGA2*, *ZAP1*, *POL31*, *RAV1*, *POL32*, *RFC2*, *NNF1*, *ASK1*, *RAD27*, *SNU114*, *SAC1*, *ORC3*, *COF1*, *RIC1*, *RPL22A*, *UPS2*, *MMR1*, *SEC22*, *MCM5*, *STT4*, *GAS2*, *TEM1*, *POB3*, *SEC59*, *CSM3*, *CEP3*, *ERG8*, *PEP5*, *LCB1*, *PSE1*, *ADE12*, *TOF1*, *ATG3*, *TLG2*, *SMC5*, *RFC4*, *BUB3*, *RPT4*, *RRS1*, *COT1*, *ERG10*, *SPC29*, *ODC1*, *IPL1*
GO:0006281	DNA repair	7.21E-04	*DPB3*, *MRC1*, *MCD1*, *PDS1*, *MMS21*, *RPT6*, *POL31*, *POL32*, *RFC2*, *RAD27*, *MCM5*, *POB3*, *CSM3*, *TOF1*, *SMC5*, *RFC4*, *RPT4*
GO:0022616	DNA strand elongation	7.93E-04	*DPB3*, *DPB4*, *POL31*, *POL32*, *RFC2*, *RAD27*, *RFC4*
GO:0044428	Nuclear part	8.03E-04	*SWH1*, *ORC2*, *ABD1*, *DPB3*, *MRC1*, *YCL075W*, *MCD1*, *APC11*, *RRI1*, *FAL1*, *DPB4*, *TAF12*, *MMS21*, *KAP122*, *CBF2*, *RPB3*, *MET30*, *POL31*, *POL32*, *NNF1*, *ASK1*, *RAD27*, *SNU114*, *ORC3*, *COF1*, *MCM5*, *POB3*, *CSM3*, *CEP3*, *TOF1*, *BUB3*, *RRS1*, *IPL1*
GO:0006996	Organelle organization	8.60E-04	*SSA1*, *SLA1*, *STU1*, *SEC17*, *PKC1*, *ORC2*, *CCZ1*, *MRC1*, *MCD1*, *APC11*, *USO1*, *TIM22*, *VPS41*, *PDS1*, *TAF12*, *SSD1*, *TSC11*, *RSP5*, *MOB2*, *RPT6*, *DBF2*, *CBF2*, *RFC2*, *NNF1*, *ASK1*, *COF1*, *UPS2*, *MMR1*, *SEC22*, *MCM5*, *TEM1*, *POB3*, *CSM3*, *CEP3*, *PEP5*, *TOF1*, *ATG3*, *TLG2*, *RFC4*, *SPC29*, *IPL1*
GO:0000777	Condensed chromosome kinetochore	1.16E-03	*STU1*, *SLI15*, *CBF2*, *NNF1*, *ASK1*, *CEP3*, *BUB3*, *IPL1*
GO:0000775	Chromosome, centromeric region	1.18E-03	*STU1*, *SLI15*, *MCD1*, *CBF2*, *NNF1*, *ASK1*, *CEP3*, *BUB3*, *IPL1*
GO:0043228	Nonmembrane-bounded organelle	1.40E-03	*SLA1*, *STU1*, *PKC1*, *ORC2*, *SLI15*, *DPB3*, *MRC1*, *MCD1*, *USO1*, *FAL1*, *PDS1*, *DPB4*, *MMS21*, *DBF2*, *CBF2*, *POL31*, *POL32*, *RFC2*, *NNF1*, *ASK1*, *RAD27*, *ORC3*, *COF1*, *RPL22A*, *MCM5*, *TEM1*, *POB3*, *CSM3*, *CEP3*, *TOF1*, *SMC5*, *RFC4*, *BUB3*, *RRS1*, *SPC29*, *IPL1*
GO:0043232	Intracellular nonmembrane-bounded organelle	1.40E-03	*SLA1*, *STU1*, *PKC1*, *ORC2*, *SLI15*, *DPB3*, *MRC1*, *MCD1*, *USO1*, *FAL1*, *PDS1*, *DPB4*, *MMS21*, *DBF2*, *CBF2*, *POL31*, *POL32*, *RFC2*, *NNF1*, *ASK1*, *RAD27*, *ORC3*, *COF1*, *RPL22A*, *MCM5*, *TEM1*, *POB3*, *CSM3*, *CEP3*, *TOF1*, *SMC5*, *RFC4*, *BUB3*, *RRS1*, *SPC29*, *IPL1*
GO:0005488	Binding	1.98E-03	*SSA1*, *SWH1*, *SLA1*, *STU1*, *URA7*, *SEC17*, *PKC1*, *ORC2*, *TEC1*, *ARL1*, *ABD1*, *DPB3*, *YCL075W*, *MCD1*, *APC11*, *USO1*, *COP1*, *RRI1*, *TIM22*, *FAL1*, *VPS41*, *PDS1*, *DPB4*, *YCF1*, *TAF12*, *SSD1*, *MFA1*, *MMS21*, *YPT31*, *TSC11*, *RSP5*, *KAP122*, *RPT6*, *PAC10*, *DBF2*, *CBF2*, *RPB3*, *MET30*, *ZAP1*, *POL31*, *RFC2*, *ASK1*, *RAD27*, *SNU114*, *ORC3*, *COF1*, *SEC22*, *MCM5*, *STT4*, *GAS2*, *TEM1*, *POB3*, *CEP3*, *ERG8*, *PEP5*, *LCB1*, *PSE1*, *ADE12*, *TLG2*, *SMC5*, *RFC4*, *BUB3*, *RPT4*, *ERG10*, *ODC1*, *IPL1*
GO:0000779	Condensed chromosome, centromeric region	2.03E-03	*STU1*, *SLI15*, *CBF2*, *NNF1*, *ASK1*, *CEP3*, *BUB3*, *IPL1*
GO:0003887	DNA-directed DNA polymerase activity	2.23E-03	*DPB3*, *YCL075W*, *DPB4*, *POL31*, *POL32*
GO:0030894	Replisome	2.25E-03	*DPB3*, *DPB4*, *POL31*, *POL32*, *POB3*
GO:0043601	Nuclear replisome	2.25E-03	*DPB3*, *DPB4*, *POL31*, *POL32*, *POB3*
GO:0000776	Kinetochore	2.32E-03	*STU1*, *SLI15*, *CBF2*, *NNF1*, *ASK1*, *CEP3*, *BUB3*, *IPL1*
GO:0016043	Cellular component organization	3.46E-03	*SSA1*, *SWH1*, *SLA1*, *STU1*, *SEC17*, *PKC1*, *ORC2*, *TEC1*, *CCZ1*, *MRC1*, *MCD1*, *APC11*, *USO1*, *TIM22*, *HBT1*, *VPS41*, *PDS1*, *TAF12*, *SSD1*, *TSC11*, *RSP5*, *MOB2*, *KAP122*, *RPT6*, *PAC10*, *DBF2*, *CBF2*, *RFC2*, *NNF1*, *ASK1*, *SNU114*, *ORC3*, *COF1*, *UPS2*, *MMR1*, *SEC22*, *MCM5*, *GAS2*, *TEM1*, *POB3*, *CSM3*, *CEP3*, *PEP5*, *TOF1*, *ATG3*, *TLG2*, *RFC4*, *RPT4*, *SPC29*, *IPL1*
GO:0031981	Nuclear lumen	3.85E-03	*ORC2*, *ABD1*, *DPB3*, *MRC1*, *MCD1*, *FAL1*, *DPB4*, *TAF12*, *CBF2*, *RPB3*, *POL31*, *POL32*, *NNF1*, *ASK1*, *RAD27*, *ORC3*, *COF1*, *MCM5*, *POB3*, *CSM3*, *CEP3*, *TOF1*, *BUB3*, *RRS1*, *IPL1*
GO:0034061	DNA polymerase activity	3.90E-03	*DPB3*, *YCL075W*, *DPB4*, *POL31*, *POL32*
GO:0000278	Mitotic cell cycle	3.93E-03	*STU1*, *MRC1*, *MCD1*, *APC11*, *PDS1*, *SSD1*, *MOB2*, *CBF2*, *MET30*, *NNF1*, *ASK1*, *MCM5*, *TEM1*, *CSM3*, *TOF1*, *BUB3*, *IPL1*
GO:0031298	Replication fork protection complex	4.09E-03	*MRC1*, *MCM5*, *POB3*, *CSM3*, *TOF1*
GO:0006259	DNA metabolic process	4.13E-03	*ORC2*, *DPB3*, *MRC1*, *MCD1*, *PDS1*, *DPB4*, *MMS21*, *RPT6*, *POL31*, *POL32*, *RFC2*, *RAD27*, *ORC3*, *MCM5*, *POB3*, *CSM3*, *TOF1*, *SMC5*, *RFC4*, *RPT4*
GO:0000280	Nuclear division	4.90E-03	*STU1*, *MRC1*, *MCD1*, *APC11*, *PDS1*, *SSD1*, *MOB2*, *DBF2*, *NNF1*, *ASK1*, *TEM1*, *CSM3*, *TOF1*
GO:0000087	M phase of mitotic cell cycle	5.63E-03	*STU1*, *MRC1*, *MCD1*, *APC11*, *PDS1*, *SSD1*, *MOB2*, *MET30*, *NNF1*, *ASK1*, *TEM1*, *CSM3*, *TOF1*
GO:0051716	Cellular response to stimulus	6.38E-03	*PKC1*, *CCZ1*, *ARL1*, *DPB3*, *MRC1*, *MCD1*, *RRI1*, *HBT1*, *PDS1*, *MFA1*, *MMS21*, *YPT31*, *TSC11*, *RSP5*, *RPT6*, *MGA2*, *ZAP1*, *POL31*, *POL32*, *RFC2*, *RAD27*, *MCM5*, *STT4*, *TEM1*, *POB3*, *CSM3*, *TOF1*, *ATG3*, *SMC5*, *RFC4*, *RPT4*
GO:0005819	Spindle	6.45E-03	*STU1*, *SLI15*, *PDS1*, *DBF2*, *CBF2*, *ASK1*, *TEM1*, *SPC29*, *IPL1*
GO:0042575	DNA polymerase complex	6.93E-03	*DPB3*, *DPB4*, *POL31*, *POL32*
GO:0007049	Cell cycle	7.00E-03	*STU1*, *PKC1*, *MRC1*, *RMD1*, *MCD1*, *APC11*, *PDS1*, *SSD1*, *MOB2*, *DBF2*, *CBF2*, *MET30*, *RFC2*, *NNF1*, *ASK1*, *MMR1*, *MCM5*, *TEM1*, *CSM3*, *TOF1*, *SMC5*, *RFC4*, *BUB3*, *SPC29*, *IPL1*
GO:0006974	Response to DNA damage stimulus	7.21E-03	*DPB3*, *MRC1*, *MCD1*, *PDS1*, *MMS21*, *RPT6*, *POL31*, *POL32*, *RFC2*, *RAD27*, *MCM5*, *POB3*, *CSM3*, *TOF1*, *SMC5*, *RFC4*, *RPT4*
GO:0048285	Organelle fission	8.96E-03	*STU1*, *MRC1*, *MCD1*, *APC11*, *PDS1*, *SSD1*, *MOB2*, *DBF2*, *NNF1*, *ASK1*, *TEM1*, *CSM3*, *TOF1*
GO:0071840	Cellular component organization or biogenesis	9.43E-03	*SSA1*, *SWH1*, *SLA1*, *STU1*, *SEC17*, *PKC1*, *ORC2*, *TEC1*, *CCZ1*, *MRC1*, *MCD1*, *APC11*, *USO1*, *TIM22*, *HBT1*, *FAL1*, *VPS41*, *PDS1*, *TAF12*, *SSD1*, *TSC11*, *RSP5*, *MOB2*, *KAP122*, *RPT6*, *PAC10*, *DBF2*, *CBF2*, *RFC2*, *NNF1*, *ASK1*, *SNU114*, *ORC3*, *COF1*, *UPS2*, *MMR1*, *SEC22*, *MCM5*, *GAS2*, *TEM1*, *POB3*, *CSM3*, *CEP3*, *PEP5*, *TOF1*, *ATG3*, *TLG2*, *RFC4*, *RPT4*, *RRS1*, *SPC29*, *IPL1*
GO:0030174	Regulation of DNA-dependent DNA replication initiation	1.22E-02	*MRC1*, *MET30*, *MCM5*, *CSM3*, *TOF1*
GO:0006273	Lagging strand elongation	2.07E-02	*DPB3*, *DPB4*, *POL31*, *POL32*, *RAD27*
GO:0007067	Mitosis	2.18E-02	*STU1*, *MRC1*, *MCD1*, *APC11*, *PDS1*, *SSD1*, *MOB2*, *NNF1*, *ASK1*, *TEM1*, *CSM3*, *TOF1*
GO:0071841	Cellular component organization or biogenesis at cellular level	2.18E-02	*SSA1*, *SLA1*, *STU1*, *SEC17*, *PKC1*, *ORC2*, *CCZ1*, *MRC1*, *MCD1*, *APC11*, *USO1*, *TIM22*, *FAL1*, *VPS41*, *PDS1*, *TAF12*, *SSD1*, *TSC11*, *RSP5*, *MOB2*, *RPT6*, *PAC10*, *DBF2*, *CBF2*, *RFC2*, *NNF1*, *ASK1*, *SNU114*, *ORC3*, *COF1*, *UPS2*, *MMR1*, *SEC22*, *MCM5*, *GAS2*, *TEM1*, *POB3*, *CSM3*, *CEP3*, *PEP5*, *TOF1*, *ATG3*, *TLG2*, *RFC4*, *RPT4*, *RRS1*, *SPC29*, *IPL1*
GO:0005856	Cytoskeleton	2.56E-02	*SLA1*, *STU1*, *PKC1*, *SLI15*, *USO1*, *PDS1*, *DBF2*, *CBF2*, *ASK1*, *COF1*, *TEM1*, *SPC29*, *IPL1*
GO:0000778	Condensed nuclear chromosome kinetochore	2.75E-02	*CBF2*, *NNF1*, *ASK1*, *CEP3*, *BUB3*, *IPL1*
GO:0015630	Microtubule cytoskeleton	2.88E-02	*STU1*, *SLI15*, *PDS1*, *DBF2*, *CBF2*, *ASK1*, *TEM1*, *SPC29*, *IPL1*
GO:0022402	Cell-cycle process	3.13E-02	*STU1*, *MRC1*, *RMD1*, *MCD1*, *APC11*, *PDS1*, *SSD1*, *MOB2*, *CBF2*, *MET30*, *RFC2*, *NNF1*, *ASK1*, *MCM5*, *TEM1*, *CSM3*, *TOF1*, *SMC5*, *RFC4*, *BUB3*, *SPC29*, *IPL1*
GO:0051128	Regulation of cellular component organization	3.14E-02	*VPS41*, *SSD1*, *TSC11*, *RSP5*, *RPT6*, *RAV1*, *ASK1*, *POB3*, *CEP3*, *PEP5*, *PSE1*, *BUB3*, *RPT4*
GO:0051233	Spindle midzone	4.08E-02	*SLI15*, *CBF2*, *IPL1*

GO, Gene Ontology.

Of note were three hits from the *ade2-min3* SGA analyses that were represented in both the nonessential and the essential mutant strain sets. These hits included the stress response kinase gene *DBF2* (Johnston and Thomas 1982), the checkpoint gene *SLI15* (Kim *et al.* 1999; Ruchaud *et al.* 2007; Sandall *et al.* 2006), and the SNARE complex gene *SEC22* (Liu and Barlowe 2002). Deletion of *DBF2* and *SLI15* in the nonessential strain set resulted in strains that produced a low level of blebbing. However, ts alleles of both strains were identified as hits in our screen. Our results suggest that aberrant alleles of either gene are more detrimental to minisatellite stability than complete loss of the gene itself. We found the opposite effect for the gene *SEC22*, which was identified as a hit when deleted but not as a ts allele. Therefore, variations in gene product expression or function appear to differentially affect *ade2-min3* minisatellite stability.

Of the 102 genes identified in our screen, only three produced a high level of blebbing (++++) for at least two of the three independent analyses that were performed (Table 3). These included the zinc transporter gene *ZRT1* (Zhao and Eide 1996), the mitochondrial transport gene *ODC1* (Palmieri *et al.* 2001), and *SAC1*, a gene encoding for a lipid phosphatase involved in protein trafficking and cell wall maintenance (reviewed in Strahl and Thorner 2007). These results indicate that aberrant cellular trafficking, such as that involved in regulating intracellular protein and zinc levels, can drastically affect the stability of a minisatellite allele in stationary phase yeast cells.

### Identification of factors that maintain the stability of the ade2-h7.5 HRAS1-associated minisatellite allele in stationary-phase yeast cells

Although the *ade2-min3* allele allows us to readily assess mutant strains for minisatellite instability, its composition does not mimic most minisatellites found within human cells. The majority of human minisatellites are composed of long repeat units that are variable in sequence composition and are significantly enriched for GC content (Vergnaud and Denoeud 2000). The *ade2-min3* minisatellite is comprised of identical repeats with only 50% GC content. Because of these differences, human minisatellites may be regulated by different factors than those associated with the *ade2-min3* allele, a short minisatellite with identical repeat units.

To examine these potential issues, we used a second minisatellite reporter (the *ade2-h7.5* allele; Figure 1B), which incorporates a portion of the complex human *HRAS1* minisatellite into the *ADE2* gene (Kelly *et al.* 2007, 2011). This tract is composed of seven-and-a-half repeat units that are 28 bp in size, is variable in base content at positions +14nt and +22nt, and has a GC content of 68% (Kelly *et al.* 2011). Similar to the *ade2-min3* assay system, insertion of the tract disrupts the *ADE2* ORF, and loss of a 28-bp repeat (or gain of two repeats) will restore the proper reading frame.

To identify the factors involved in regulating the stability of a complex human-associated minisatellite during stationary phase, we used the same SGA methodology described previously, screening the strain set three independent times. A caveat to using the *ade2-h7.5* allele as a query strain for this screen is that the overall level of blebbing associated with this allele is approximately fourfold lower than that produced by *ade2-min3* strains (Kelly *et al.* 2011); this increase in minisatellite stability makes phenotypic scoring more difficult.

We identified 157 candidate hits that result in a high level of blebbing when mutated (Table 5) and performed GO term analysis as described previously. Intriguingly, only one enriched GO term (GO:0016831 carboxy-lyase activity) was associated with this data set. Within this particular set of 157 hits, we found that four mutant strains maintained a very high level of blebbing throughout our *ade2-h7.5* SGA analyses (++++ for at least two of the three screens performed; Table 5). These included the previously characterized zinc transporter genes *ZRT1* and *ZAP1* (Kelly *et al.* 2007, 2011; Zhao and Eide 1996, 1997), plus the uncharacterized gene encoded by the open reading frame YHR022C and the mismatch repair associated gene, *PMS1* (Prolla *et al.* 1994a,b). We conclude that zinc homeostasis plays an important role in preventing *ade2-h7.5* minisatellite instability and that mismatch repair influences minisatellite stability in stationary-phase cells.

**Table 5:** Summary of hits from the *ade2-h7.5* SGA analysis of the yeast nonessential strain set

Gene	ORF	Gene	ORF	Gene	ORF	Gene	ORF
*ACA1*	YER045c	*KTI12*	YKL110c	*SDS24*	YBR214w	YGL217C	YGL217c
*ACF4*	YJR083c	*LCB4*	YOR171c	*SKI8*	YGL213c	YGL230C	YGL230c
*ADY4*	YLR227c	*LEE1*	YPL054w	*SLK19*	YOR195w	YGR001C	YGR001c
*AGP3*	YFL055w	*LEU3*	YLR451w	*SNF6*	YHL025w	YGR031W	YGR031w
*AIM23*	YJL131c	*LIP5*	YOR196c	*SOL3*	YHR163w	YGR051C	YGR051c
*ALR2*	YFL050c	*MET22*	YOL064c	*SPE1*	YKL184w	YGR149W	YGR149w
*APM4*	YOL062c	*MON1*	YGL124c	*SPS22*	YCL048w	YGR176W	YGR176w
*APS1*	YLR170c	*MPC54*	YOR177c	*SRN2*	YLR119w	YGR207C	YGR207c
*ARN2*	YHL047c	*MRM2*	YGL136c	*SRX1*	YKL086w	YGR266W	YGR266w
*ART5*	YGR068c	*MRPL22*	YNL177c	*SSH4*	YKL124w	YHL044W	YHL044w
*ATO3*	YDR384c	*NCE102*	YPR149w	*SWT21*	YNL187w	**YHR022C**	**YHR022c**
*AVT3*	YKL146w	*NFI1*	YOR156c	*THI72*	YOR192c	YJL049W	YJL049w
*BMH2*	YDR099w	*NKP1*	YDR383c	*THP1*	YOL072w	YKL070W	YKL070w
*BSC1*	YDL037c	*NNK1*	YKL171w	*TIM21*	YGR033c	YKL136W	YKL136w
*BUD2*	YKL092c	*NUP2*	YLR335w	*TMT1*	YER175c	YKL151C	YKL151c
*BUD28*	YLR062c	*OXP1*	YKL215c	*TPK3*	YKL166c	YKL187C	YKL187c
*CAR1*	YPL111w	*PAR32*	YDL173w	*TPO2*	YGR138c	YLR125W	YLR125w
*COT1*	YOR316c	*PDC5*	YLR134w	*TRP4*	YDR354w	YLR225C	YLR225c
*CUE2*	YKL090w	*PDR12*	YPL058c	*TUM1*	YOR251c	YML053C	YML053c
*CYC7*	YEL039c	*PFA4*	YOL003c	*UBC11*	YOR339c	YML089C	YML089c
*DFG5*	YMR238w	*PIR3*	YKL163w	*UTR2*	YEL040w	YMR010W	YMR010w
*DGR2*	YKL121w	*PLB1*	YMR008c	*VBA4*	YDR119w	YMR085W	YMR085w
*DLD1*	YDL174c	*PMP2*	YEL017c-a	*VMA21*	YGR105w	YMR090W	YMR090w
*EAP1*	YKL204w	***PMS1***	**YNL082w**	*VMS1*	YDR049w	YMR258C	YMR258c
*EDC3*	YEL015w	*PRM4*	YPL156c	*VPS61*	YDR136c	YMR304C-A	YMR304c-a
*EFT2*	YDR385w	*PSD2*	YGR170w	*VTC1*	YER072w	YOL024W	YOL024w
*ELC1*	YPL046c	*PXL1*	YKR090w	*YAP1801*	YHR161c	YOL079W	YOL079w
*FDC1*	YDR539w	*QCR10*	YHR001w-a	*YAT2*	YER024w	YOL153C	YOL153c
*FIN1*	YDR130c	*RDS1*	YCR106w	YBL096C	YBL096c	YOR170W	YOR170w
*FOB1*	YDR110w	*REC104*	YHR157w	YBR197C	YBR197c	YOR296W	YOR296w
*GET2*	YER083c	*RGP1*	YDR137w	YBR277C	YBR277c	YPL066W	YPL066w
*GIT1*	YCR098c	*RNH203*	YLR154c	YCR015C	YCR015c	YPL102C	YPL102c
*HIR1*	YBL008w	*RPL7B*	YPL198w	YDR109C	YDR109c	YPR078C	YPR078c
*HIS6*	YIL020c	*RPS25B*	YLR333c	YDR415C	YDR415c	YPR109W	YPR109w
*HNT3*	YOR258w	*RRD1*	YIL153w	YEL020C	YEL020c	*YPT35*	YHR105w
*HOR2*	YER062c	*RRT2*	YBR246w	YEL023C	YEL023c	***ZAP1***	**YJL056c**
*HSP82*	YPL240c	*RSM22*	YKL155c	YER067C-A	YER067C-A	***ZRT1***	**YGL255w**
*IRC18*	YJL037w	*RUB1*	YDR139c	YER068C-A	YER068C-A		
*KES1*	YPL145c	*RVS167*	YDR388w	YFH7	YFR007w		
*KIN82*	YCR091w	*SAP185*	YJL098w	YGL199C	YGL199c		

Genes appearing in bold typeface are the strongest hits from the *ade2-h7.5* screen (scored as ++++ for at least two of three independent screens). GO, Gene Ontology.

### Analysis of factors involved in maintaining the stability of both the ade2-min3 and ade2-h7.5 minisatellite alleles in stationary-phase cells

**Figure 2:**
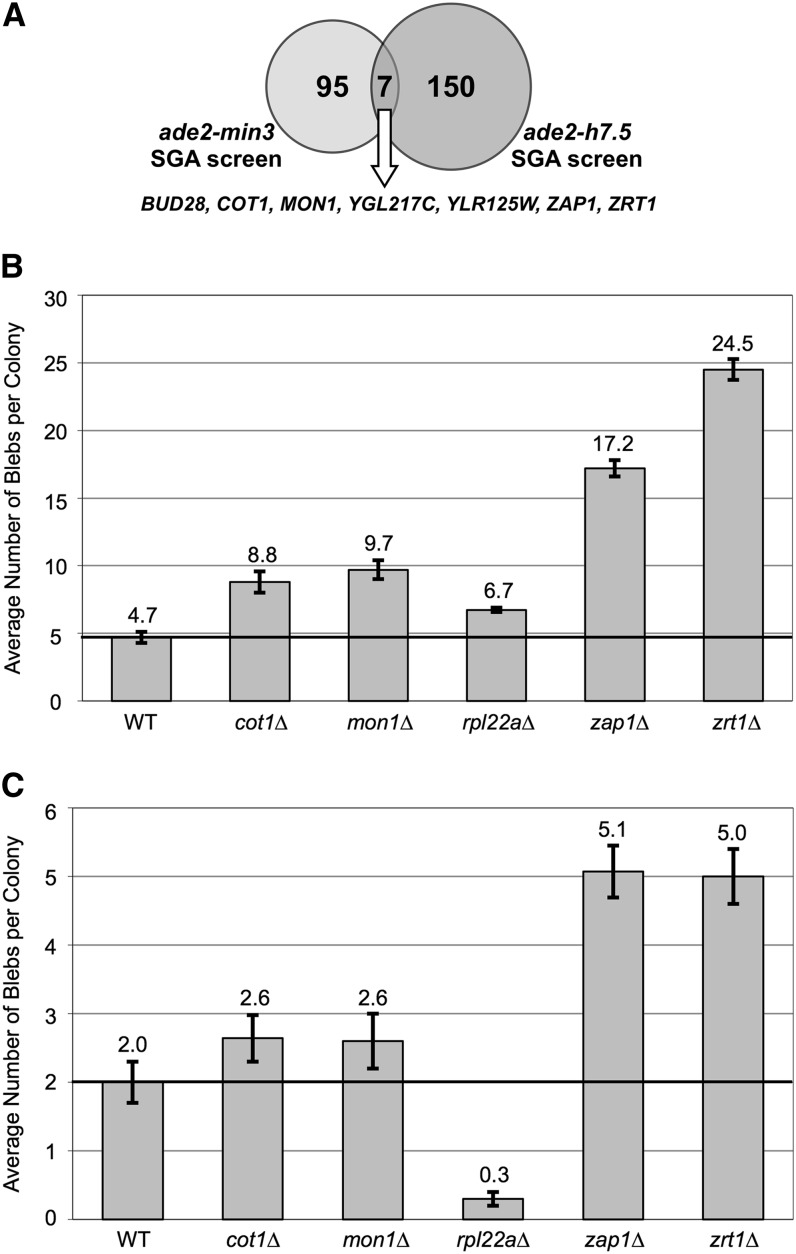
Summary of overlapping hits from the *ade2-min3* and *ade2-h7.5* SGA Screens. (A) Of the 102 hits obtained from the *ade2-min3* SGA analysis and 157 hits obtained from the *ade2-h7.5* SGA analysis, seven hits overlapped both screens. (B) Blebbing quantification of strains bearing the *ade2-min3* allele. YPD cultures were inoculated with a single red colony and grown for 4 h at 30°. Each culture was diluted and plated onto solid YPD media. Strains were incubated at 30° for 2 d and then at RT for 6 d. Blebs were counted on 100 colonies. The average number of blebs +/− the 95% confidence interval were calculated for each strain. This experiment was repeated three independent times. (C) Blebbing quantification of strains bearing the *ade2-h7.5* allele was performed as in (B).

Although the overlap of candidate hits between the *ade2-min3* and the *ade2-h7.5* screens was statistically significant (*P* = 0.047), only seven genes were identified as hits in both SGA screens (Figure 2A): the genes *BUD28*, *COT1*, *MON1*, *YGL217C*, *YLR125W*, *ZAP1*, and *ZRT1*. *BUB28* and *YGL217C* are both dubious ORFs that are unlikely to code for a protein. Each ORF, however, overlaps the coding sequence of an adjacent characterized gene. Approximately 98% of the *BUD28* ORF overlaps the ribosomal subunit gene *RPL22A* (Planta and Mager 1998) whereas *YGL217C* overlaps roughly 10% of *KIP3*, a gene that encodes for a kinesin-like protein involved in mitotic spindle positioning (Cottingham and Hoyt 1997). *RPL22A* was identified as a hit in our *ade2-min3* SGA screen but not in the *ade2-h7.5* screen. Nevertheless, it is possible that this gene was not identified in the *ade2-h7.5* screen due to the difficulty in scoring each strain. Deletion of the ORF *YGL217C* is represented twice in the nonessential deletion haploid set; one deletion strain was identified as a hit in both screens, and the second was not. No other hits were duplicated within our strain sets. PCR analysis revealed that both of the *YGL217C* isolates from the strain collection and our SGA analyses are correct deletion mutants. Therefore, one or both isolates could contain a secondary mutation that enhances or suppresses the blebbing phenotype.

PCR analysis of the *YLR125W* deletion mutant from the nonessential haploid strain set as well as of the *ade2-min3 YLR125W* mutant from the final step of the SGA analysis revealed that each strain was wild-type (WT) at the *YLR125W* locus. Because both strains are G418^R^, the *KANMX* PCR product used to construct the deletion collection parental strain must be located elsewhere in the genome, likely at an area of homology to the *YLR125W* locus. Future determination of the deletion’s genomic location may give insight into the source of blebbing observed in this strain.

The remaining genes present in each data set are associated with intracellular transport. Included are the vacuolar transport gene *MON1* and the zinc-mediating vacuolar transport gene *COT1* (Conklin *et al.* 1992; MacDiarmid *et al.* 2000; Meiling-Wesse *et al.* 2002; Wang *et al.* 2002). The final two overlapping hits are the zinc transport gene *ZRT1* and the transcriptional regulator of *ZRT1*, *ZAP1* (Zhao and Eide 1996, 1997) that were previously shown by our laboratory to be important in minisatellite stability (Kelly *et al.* 2007, 2011). These four may influence minisatellite stability in the same manner, as intracellular zinc is sequestered in vacuoles until needed. Our data suggest that the disruption of cellular transport within stationary phase cells, particularly that associated with zinc transport, results in the destabilization of a minisatellite regardless of tract length or repeat unit sequence composition.

To determine whether any sets of genes were overrepresented in both the *ade2-min3* and *ade2-h7.5* hit lists, we performed GO term analyses on the combined results of each screen (Table 6). Each enriched GO term was primarily associated with genes identified in the *ade2-min3* screen rather than those of the *ade2-h7.5* screen. Therefore, the GO terms represented general terms associated with cellular chromosomes and DNA replication as discussed previously. Based upon the low number of overlapping hits between each screen and the results from our GO term analyses, we conclude that each minisatellite tract is regulated by a distinct set of genes that do not share many overlapping functions or processes.

**Table 6:** Enriched GO terms of hits from the ade2-min3 SGA analysis of the yeast nonessential and essential strain sets and the *ade2-h7.5* SGA analysis of the yeast nonessential strain set

GO ID	GO term	*P* value	Genes
GO:0006261	DNA-dependent DNA replication	1.09E-04	*ORC2*, *DPB3*, *MRC1*, *BMH2*, *DPB4*, *POL31*, *POL32*, *RFC2*, *RAD27*, *ORC3*, *RNH203*, *MCM5*, *POB3*, *CSM3*, *TOF1*, *RFC4*
GO:0005657	Replication fork	1.04E-03	*DPB3*, *MRC1*, *DPB4*, *POL31*, *POL32*, *RFC2*, *MCM5*, *POB3*, *CSM3*, *TOF1*, *RFC4*
GO:0006272	Leading strand elongation	3.15E-03	*DPB3*, *DPB4*, *POL31*, *POL32*, *RFC2*, *RFC4*
GO:0044427	Chromosomal part	3.80E-03	*HIR1*, *STU1*, *ORC2*, *SLI15*, *DPB3*, *MRC1*, *MCD1*, *DPB4*, *FIN1*, *NKP1*, *MMS21*, *CBF2*, *RRD1*, *POL31*, *POL32*, *RFC2*, *NNF1*, *ASK1*, *ORC3*, *MCM5*, *NUP2*, *POB3*, *CSM3*, *CEP3*, *TOF1*, *SMC5*, *RFC4*, *BUB3*, *NFI1*, *SLK19*, *IPL1*
GO:0000775	Chromosome, centromeric region	4.77E-03	*HIR1*, *STU1*, *SLI15*, *MCD1*, *FIN1*, *NKP1*, *CBF2*, *NNF1*, *ASK1*, *CEP3*, *BUB3*, *SLK19*, *IPL1*
GO:0005694	Chromosome	5.50E-03	*HIR1*, *STU1*, *ORC2*, *SLI15*, *DPB3*, *MRC1*, *MCD1*, *DPB4*, *FIN1*, *NKP1*, *MMS21*, *SKI8*, *CBF2*, *REC104*, *RRD1*, *POL31*, *POL32*, *RFC2*, *NNF1*, *ASK1*, *ORC3*, *MCM5*, *NUP2*, *POB3*, *CSM3*, *CEP3*, *TOF1*, *SMC5*, *RFC4*, *BUB3*, *NFI1*, *SLK19*, *IPL1*
GO:0000777	Condensed chromosome kinetochore	7.00E-03	*STU1*, *SLI15*, *FIN1*, *NKP1*, *CBF2*, *NNF1*, *ASK1*, *CEP3*, *BUB3*, *SLK19*, *IPL1*
GO:0043596	Nuclear replication fork	9.84E-03	*DPB3*, *MRC1*, *DPB4*, *POL31*, *POL32*, *MCM5*, *POB3*, *CSM3*, *TOF1*
GO:0006260	DNA replication	1.30E-02	*ORC2*, *DPB3*, *MRC1*, *BMH2*, *DPB4*, *POL31*, *POL32*, *RFC2*, *RAD27*, *ORC3*, *RNH203*, *MCM5*, *POB3*, *CSM3*, *TOF1*, *RFC4*
GO:0000779	Condensed chromosome, centromeric region	1.43E-02	*STU1*, *SLI15*, *FIN1*, *NKP1*, *CBF2*, *NNF1*, *ASK1*, *CEP3*, *BUB3*, *SLK19*, *IPL1*
GO:0000776	Kinetochore	1.69E-02	*STU1*, *SLI15*, *FIN1*, *NKP1*, *CBF2*, *NNF1*, *ASK1*, *CEP3*, *BUB3*, *SLK19*, *IPL1*
GO:0000793	Condensed chromosome	1.76E-02	*STU1*, *SLI15*, *MCD1*, *FIN1*, *NKP1*, *MMS21*, *CBF2*, *REC104*, *NNF1*, *ASK1*, *CEP3*, *SMC5*, *BUB3*, *SLK19*, *IPL1*
GO:0006271	DNA strand elongation involved in DNA replication	3.26E-02	*DPB3*, *DPB4*, *POL31*, *POL32*, *RFC2*, *RAD27*, *RNH203*, *RFC4*
GO:0051233	Spindle midzone	3.53E-02	*SLI15*, *CBF2*, *SLK19*, *IPL1*
GO:0005819	Spindle	4.39E-02	*STU1*, *SLI15*, *PDS1*, *FIN1*, *DBF2*, *CBF2*, *ASK1*, *ADY4*, *TEM1*, *MPC54*, *SLK19*, *SPC29*, *IPL1*

GO, Gene Ontology; SGA, synthetic genetic array.

To verify the results of the SGA analysis, we deleted these genes in a separate genetic background—our well-characterized *ade2-min3* (DTK271) and *ade2-h7.5* (DTK1188) strain background (Alver *et al.* 2013; Kelly *et al.* 2007, 2011). We then quantified the blebbing phenotype in the resulting mutants. As previously reported, deletion of *ZAP1* and *ZRT1* in both *ade2-min3* and *ade2-h7.5* strain backgrounds resulted in a high level of blebbing compared with the WT parental strains (Figure 2, B and C) (Kelly *et al.* 2007, 2011). However, unlike the results from the SGA analyses, strains bearing a deletion of *COT1*, *MON1*, or *RPL22A* did not result in a dramatic increase in blebbing in either strain background. The deletion of *COT1*, *MON1*, or *RPL22A* in a strain bearing the *ade2-min3* allele produced a level of blebbing that, although significantly greater than that of the WT strain, was only 30% of that displayed by the *zrt1*Δ strain (*cot1*Δ at 8.8 blebs/colony; *mon1*Δ at 9.7 blebs/colony; *rpl22a*Δ at 6.7 blebs/colony *vs.* WT at 4.7 blebs/colony and *zrt1*Δ at 24.5 blebs/colony). An *ade2-h7.5* strain bearing a deletion of *COT1*, *MON1*, or *RPL22A* did not produce a significant increase in blebbing compared with the WT strain (*cot1*Δ at 2.6 blebs/colony; *mon1*Δ at 2.6 blebs/colony; *rpl22a*Δ at 0.3 blebs/colony *vs.* WT at 2.0 blebs/colony). We suspect that these results could be due to a secondary mutation in the SGA strain background that could act as an enhancer, or in our laboratory strain background which could act as a suppressor of the blebbing phenotype.

### Mismatch repair regulates ade2-h7.5 minisatellite stability in stationary-phase cells

**Figure 3:**
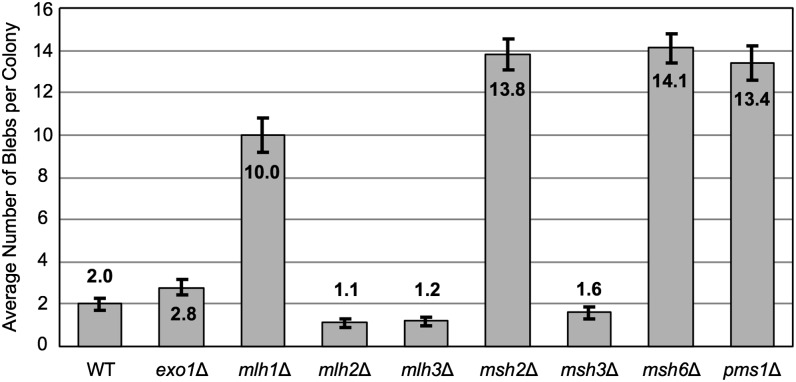
Specific mismatch repair components stabilize minisatellites in stationary phase. Blebbing quantification for *ade2-h7.5* strains bearing a deletion of a nonessential mismatch repair gene was performed as described in Figure 2 and in *Materials and Methods*.

Previous work in actively dividing cells suggested that mismatch repair is associated with preventing microsatellite, rather than minisatellite, alterations (Sia *et al.* 1997). However, the deletion of *PMS1* in the *ade2-h7.5* strain resulted in a strong blebbing phenotype, indicating a potential role for mismatch repair in stationary phase cells (Table 5). *PMS1* encodes a mismatch repair protein that, together with Mlh1p, repairs multiple forms of damaged DNA (Prolla *et al.* 1994a, b). To verify the results of our screen, we deleted *PMS1* in our laboratory strain background (Kelly *et al.* 2011) and quantified the average number of blebs/colony. Deletion of *PMS1* confirmed the results from the SGA analysis, as *pms1*Δ produced a significantly higher level of blebbing (13.4 blebs/colony) compared with the WT strain (2.0 blebs/colony; Figure 3).

To determine whether other well-characterized mismatch repair genes (reviewed in Marti *et al.* 2002) were involved in maintaining stationary phase minisatellite stability, we quantified the level of blebbing in several mismatch repair mutant strains bearing the *ade2-h7.5* allele (Figure 3). Strains with a deletion of *MLH1* (10.0 blebs/colony), *MSH2* (13.8 blebs/colony), or *MSH6* (14.1 blebs/colony) produced notably greater levels of blebbing compared with the WT strain. Deletion of *EXO1*, *MLH2*, *MLH3*, or *MSH3* did not result in a level of blebbing significantly different from that of the WT strain. Together our results indicate that a specific subset of mismatch repair genes maintains the stationary phase stability of the variable-repeat *HRAS1*- associated minisatellite. The *MLH1*, *MSH2*, and *MSH6* results also demonstrate that our SGA screens may not have identified all genes that contribute to minisatellite stability, possibly due to issues of sensitivity or strain background.

## Discussion

We used a modified version of the SGA procedure (Alver *et al.* 2013; Li *et al.* 2011; Tong and Boone 2006; Tong *et al.* 2001) to identify genes involved in maintaining minisatellite stability in stationary phase cells. We performed two individual screens; the first screen used a query strain bearing a minisatellite consisting of three identical 20-bp repeats (*ade2-min3* allele) (Kelly *et al.* 2007, 2011, 2012), whereas the second screen utilized a query strain containing a minisatellite consisting of seven-and-a-half 28-bp repeats of the *HRAS1*-associated minisatellite allele (*ade2-h7.5* allele) (Kelly *et al.* 2007, 2011). Each screen incorporated analysis of approximately 4800 nonessential genes with an additional 450 essential genes screened using the *ade2-min3* allele. We identified 102 genes that are involved in regulating the stability of the *ade2-min3* minisatellite and 157 genes that regulate the stability of the *ade2-h7.5* minisatellite in stationary phase cells. Only seven hits overlapped both screens. Finally, we demonstrated that mismatch repair genes regulate *ade2-h7.5* minisatellite stability.

We investigated the hits of each screen independently to characterize candidate genes associated with each individual minisatellite. Several hits identified in the *ade2-min3* screen were genes associated with checkpoint function and were described previously (Alver *et al.* 2013). Other identified genes involve DNA replication and repair. These included *POL31*, a subunit of Polδ (Giot *et al.* 1997; Hashimoto *et al.* 1998; Sugimoto *et al.* 1995) and the Polε subunits *DPB3* or *DPB4* (Araki 1994; Araki *et al.* 1991; Lou *et al.* 2008) (Table 3). Also included were the *RFC2* and *RFC4* subunits of replication factor C (Cullmann *et al.* 1995; Noskov *et al.* 1994; Yao *et al.* 2003). Replication factor C is a clamp loader of the proliferating cell nuclear antigen, a sliding clamp for Polδ and Polε (Chilkova *et al.* 2007). Both Polδ and Polε have been implicated in DNA repair mechanisms. Polδ has been shown to be involved in base excision repair (Blank *et al.* 1994), repair of UV-damaged DNA (Torres-Ramos *et al.* 1997), and template switching after DNA damage occurs (Vanoli *et al.* 2010). Polε has been implicated in nucleotide excision repair (Shivji *et al.* 1995), base excision repair (Wang *et al.* 1993), and double-strand break repair (Holmes and Haber 1999). GO term analysis of the 102 hits from the *ade2-min3* screen revealed that the majority of enriched GO terms were associated with chromosomal regulation and DNA replication. Although it might seem surprising to find such strong evidence of DNA replication in stationary-phase cells, as bulk DNA synthesis does not take place within this population, previous work has shown that discrete areas of DNA replication do occur, likely at regions of localized DNA repair (de Morgan *et al.* 2010). Our results suggest that DNA replication is involved in preventing minisatellite alterations in stationary phase, potentially at sites of genomic repair.

Additional replication and repair genes identified in our screen included the sumo-ligase gene *MMS21* (Montelone and Koelliker 1995; Prakash and Prakash 1977) and *RAD27*, a flap endonuclease involved in base excision repair and double-strand break repair (Tseng and Tomkinson 2004; Wu *et al.* 1999). *RAD27* has previously been implicated in minisatellite stability by a number of research groups (Kelly *et al.* 2012; Lopes *et al.* 2002). Together, our data suggest that components of DNA replication and repair mechanisms prevent *ade2-min3* minisatellite alterations in stationary phase cells.

GO term analysis of candidate hits from the *ade2-h7.5* SGA analysis showed that only one term (carboxy-lyase activity) was overrepresented within our data set. This finding could be a consequence of factors that regulate the stability of the *ade2-h7.5* minisatellite representing a wide range of cellular functions, or the inherent low level of blebbing associated with the *ade2-h7.5* allele (Kelly *et al.* 2011) complicating the accuracy of scoring this screen. Also, it is possible that uncharacterized candidate hits could be specific to stationary phase gene expression, but a comparison of the uncharacterized hits with genes known to be expressed in stationary phase cells revealed no strong correlation (Aragon *et al.* 2008; Davidson *et al.* 2011).

Interestingly, only seven gene hits overlapped between the *ade2-min3* and *ade2-h7.5* screens (Figure 2A): *BUD28*, *COT1*, *MON1*, *YGL217C*, *YLR125W*, *ZAP1*, and *ZRT1*. Deletion of each gene in our laboratory strain background revealed that, like the SGA analyses, deletion of *ZRT1* or *ZAP1* resulted in a dramatic increase in minisatellite instability in both the *ade2-min3* and *ade2-h7.5* alleles (Kelly *et al.* 2007, 2011). Deletion of *COT1*, *MON1*, or *RPL22A* (*BUD28*) led to a moderate increase in *ade2-min3* instability but did not affect *ade2-h7.5* instability. Thus, differences in strain backgrounds, such as the presence of a secondary enhancer or suppressor mutation, can significantly affect minisatellite stability in stationary phase cells. In support of this, the nonessential gene deletion strain collection previously has been shown to harbor secondary mutations (Lehner *et al.* 2007). The small degree of overlap between the screens further suggests that sequence differences between the *ade2-min3* and *ade2-h7.5* minisatellites may govern repeat tract stability and alteration. Our laboratory, as well as others, has previously shown that tract length and sequence variability within a minisatellite allele can greatly affect repeat stability (Denoeud *et al.* 2003; Jauert and Kirkpatrick 2005; Legendre *et al.* 2007). This study supports these findings and extends it to stationary phase cells, suggesting that the composition and size of the minisatellite affect which cellular components and mechanisms govern tract stability.

Surprisingly, a strong candidate hit identified in the *ade2-h7.5* screen was the mismatch repair gene *PMS1* (Prolla *et al.* 1994a,b). The mismatch repair system has usually been associated with instability in microsatellite tracts, rather than minisatellite tracts (Johnson *et al.* 1996; Strand *et al.* 1993, 1995). Minisatellite instability in actively dividing cells was not significantly affected by mutations in mismatch repair mutant strains (Sia *et al.* 1997), in an analysis that used a minisatellite tract identical to the *ade2-min3* allele. Deletion of *PMS1* affected only the stability of the *ade2-h7.5* minisatellite allele rather than the *ade2-min3* allele. Only a subset of the mismatch repair components (*PMS1*, *MLH1*, *MSH2*, and *MSH6*) affected the stability (Figure 3) of the *ade2-h7.5* minisatellite tract. Previous work in actively growing cells demonstrated that mutating the mismatch repair genes *MLH1*, *MSH2* or *PMS1* resulted in a high degree of microsatellite instability, while mutating *MSH3* or *MSH6* had a less drastic effect (Johnson *et al.* 1996; Sia *et al.* 1997; Strand *et al.* 1993, 1995). This and other work suggested that yeast contain distinct mismatch repair complexes; one complex contains the Mhl1-Pms1 and Msh2-Msh6 heterodimers, whereas another incorporates Mlh1-Pms1 and Msh2-Msh3 (Johnson *et al.* 1996; Marsischky *et al.* 1996). Each complex has been implicated in the repair of different substrates; the Msh2-Msh6 heteromer is involved in base:base mismatch repair as well as repair of single-base loops generated by insertion or deletion mispairing (Alani 1996; Marsischky *et al.* 1996) whereas Msh2-Msh3 primarily targets small loops (Habraken *et al.* 1996; Marsischky *et al.* 1996). Based upon our data, we predict that the Mlh1-Pms1, Msh2-Msh6 complex prevents the instability of the *ade2-h7.5* minisatellite by targeting and repairing mismatches that can occur at two positions within a repeat. If there is misalignment of repeat units during recombinational repair (shown to be required for tract alterations) (Kelly *et al.* 2011), there will be potential C/C or G/G mispairs at nucleotides 14 and 22 in each repeat; these would be substrates for mismatch repair that could also affect tract length during the repair process. Our results lend further support to the idea that the composition of a minisatellite may dictate which factors are involved in preventing tract alterations within stationary phase cells.

In summary, we have conducted the first whole-genome screens to identify factors that regulate minisatellite stability in stationary phase cells, uncovering more than 250 genes that strongly affect the stability of minisatellite tracts in stationary-phase cells. We provide evidence that factors involved in regulating stationary-phase minisatellite stability are affected by minisatellite repeat length or sequence, as we found only a small overlap in detected genes when using significantly different minisatellite tracts. We find that disruption of DNA replication and repair components result in a dramatic increase in instability of a simple minisatellite tract, whereas loss of a subset of mismatch repair proteins specifically influences variable-repeat minisatellite instability. Thus, our work lends support to the argument that the composition of the repeat tract within a minisatellite greatly affects minisatellite stability and regulatory mechanisms.

## Supplementary Material

Supporting Information
