# Supplementary material for: A Whole Genome Screen for Minisatellite Stability Genes in Stationary-Phase Yeast Cells
Source: G3 (Bethesda). 2013 Apr 1;3(4):741–56. doi: 10.1534/g3.112.005397 (PMC3618361; doi:10.1534/g3.112.005397)
Supplement: Supporting Information [file supp_3_4_741__index.html]

A Whole Genome Screen for Minisatellite Stability Genes in Stationary-Phase Yeast Cells — Supporting Information 

# A Whole Genome Screen for Minisatellite Stability Genes in Stationary-Phase Yeast Cells

## Supporting Information for Alver *et al.*, 2013

**Files in this Data Supplement:**

- Supporting Information - Files S1-S3 (PDF, 84 KB)
- File S1 - *ade2-min3* Nonessential Strain Set SGA Blebbing Scores (.xls, 508 KB)
- File S2 - *ade2-h7.5* Nonessential Strain Set SGA Blebbing Scores (.xls, 514 KB)
- File S3 - *ade2-min3* Essential ts Allele Strain Set Blebbing Scores (.xls, 64 KB)
