# Supplementary material for: A Whole Genome Screen for Minisatellite Stability Genes in Stationary-Phase Yeast Cells
Source: G3 (Bethesda). 2013 Apr 1;3(4):741–56. doi: 10.1534/g3.112.005397 (PMC3618361; doi:10.1534/g3.112.005397)
Supplement: Supporting Information [file supp_g3.112.005397_005397SI.pdf]

G3· Genes | Genomes | Genetics

A Whole Genome Screen for Minisatellite Stability Genes in Stationary Phase Yeast Cells

Bonnie Alver*[1]

Peter A. Jauert*[1]

Laura Brosnan*

Melissa O'Hehir*

Benjamin VanderSluis[§]

Chad L. Myers[§]

David T. Kirkpatrick[2]*

*Department of Genetics, Cell Biology and Development

[§]Department of Computer Science and Engineering

University of Minnesota

Minneapolis, MN 55455

DOI: 10.1534/g3.112.005397

Files S1-S3
Supporting Data

Available for download at http://www.g3journal.org/lookup/suppl/doi:10.1534/g3.112.005397/-/DC1**.**

File S1 *ade2-min3* Nonessential Strain Set SGA Blebbing Scores

File S2 *ade2-h7.5* Nonessential Strain Set SGA Blebbing Scores

File S3 *ade2-min3* Essential ts Allele Strain Set Blebbing Scores
